# Protein Quality and the Protein to Carbohydrate Ratio within a High Fat Diet Influences Energy Balance and the Gut Microbiota In C57BL/6J Mice

**DOI:** 10.1371/journal.pone.0088904

**Published:** 2014-02-10

**Authors:** Liam McAllan, Peter Skuse, Paul D. Cotter, Paula O' Connor, John F. Cryan, R. Paul Ross, Gerald Fitzgerald, Helen M. Roche, Kanishka N. Nilaweera

**Affiliations:** 1 Food Biosciences Department, Teagasc, Fermoy, County Cork, Ireland; 2 Department of Pharmacology and Therapeutics, University College Cork, Cork, Ireland; 3 Department of Microbiology, University College Cork, Cork, Ireland; 4 Alimentary Pharmabiotic Centre, University College Cork, Cork, Ireland; 5 Department of Anatomy & Neuroscience, University College Cork, Cork, Ireland; 6 UCD Conway Institute of Biomolecular & Biomedical Research, University College Dublin, Dublin, Ireland; Pennington Biomed Research Center, United States of America

## Abstract

Macronutrient quality and composition are important determinants of energy balance and the gut microbiota. Here, we investigated how changes to protein quality (casein versus whey protein isolate; WPI) and the protein to carbohydrate (P/C) ratio within a high fat diet (HFD) impacts on these parameters. Mice were fed a low fat diet (10% kJ) or a high fat diet (HFD; 45% kJ) for 21 weeks with either casein (20% kJ, HFD) or WPI at 20%, 30% or 40% kJ. In comparison to casein, WPI at a similar energy content normalised energy intake, increased lean mass and caused a trend towards a reduction in fat mass (*P* = 0.08), but the protein challenge did not alter oxygen consumption or locomotor activity. WPI reduced HFD-induced plasma leptin and liver triacylglycerol, and partially attenuated the reduction in adipose FASN mRNA in HFD-fed mice. High throughput sequence-based analysis of faecal microbial populations revealed microbiota in the HFD-20% WPI group clustering closely with HFD controls, although WPI specifically increased *Lactobacillaceae/Lactobacillus* and decreased *Clostridiaceae/Clostridium* in HFD-fed mice. There was no effect of increasing the P/C ratio on energy intake, but the highest ratio reduced HFD-induced weight gain, fat mass and plasma triacylglycerol, non-esterified fatty acids, glucose and leptin levels, while it increased lean mass and oxygen consumption. Similar effects were observed on adipose mRNA expression, where the highest ratio reduced HFD-associated expression of UCP-2, TNFα and CD68 and increased the diet-associated expression of β3-AR, LPL, IR, IRS-1 and GLUT4. The P/C ratio also impacted on gut microbiota, with populations in the 30/40% WPI groups clustering together and away from the 20% WPI group. Taken together, our data show that increasing the P/C ratio has a dramatic effect on energy balance and the composition of gut microbiota, which is distinct from that caused by changes to protein quality.

## Introduction

It is widely recognised that levels of obesity and related clinical conditions such as diabetes, stroke, hyperlipidemia and cardiovascular disease are increasing worldwide [1]. Importantly, the development of obesity increases the set point at which the body weight, more specifically body fat, is defended, thus making its reversal difficult to achieve [2], [3]. As such, there is an increased research interest to develop effective treatments for this disease.

Dairy proteins belonging to the whey fraction (a by-product of cheese manufacture) have been increasingly tested for their potential anti-obesity effect, specifically for their ability to reduce high fat diet (HFD)-associated body weight and fat mass gain [4]–[6]. Shi *et al.,* [7] showed that replacing 5%, 50% or 100% of the dietary casein protein-derived energy content with a lactoperoxidase and lactoferrin-enriched whey protein isolate (WPI) caused a proportional suppression of body weight gain in HFD fed mice. We have previously demonstrated that a WPI-related reduction in body weight and fat mass gain in HFD fed mice was accompanied by a normalisation of energy intake and complete or partial reversal of energy balance-related gene expression in the adipose tissue and the hypothalamus [8]. While these data suggest that whey proteins have specific-effects on energy balance, such effects appear be modified by the macronutrient composition in the diet [9]. In the latter study, it was shown that increasing the lipid to carbohydrate ratio within a whey protein-rich diet significantly reduced energy intake and bodyweight gain in rats. Collectively, these data suggest that protein quality and macronutrient composition are important determinants of energy balance.

Interestingly, diet is also an important factor in determining the composition of the gut microbiota [10], [11] and specific gut microbiota signatures are associated with obesity phenotypes in animals and humans [12]–[14]. Notably, studies have shown specific whey proteins to possess anti-microbial activity [15]–[17], and that the digestive process itself facilitates the formation of potent antimicrobial whey-derived peptides, such as pepsin catalysed lactoferrin to lactoferricin [18]. A study by Sprong *et al.,* [19] demonstrated that in comparison to casein, whey protein intake increased levels of lactobacilli and bifidobacteria in a rat model of colitis. However, in a more recent study, whey protein intake was found to have no influence on gut microbiota composition in mice fed a HFD for 7 or 13 weeks [20]. Several key unanswered questions are; could whey proteins specifically influence the gut microbiota composition associated with prolonged high fat feeding, and would any changes relate to energy balance? Could changes to protein to carbohydrate ratio within a HFD vary the gut microbiota profile and energy balance in a different way to changes to protein quality?

To assess WPI specific effects on above parameters, we subjected male C57BL/6J mice to 21 weeks of either a low fat diet (LFD) with 20% kJ casein or a HFD with 20% kJ casein or WPI. In addition, using two additional HFD dietary groups on 30 or 40% kJ WPI, we evaluated the impact of increasing the protein to carbohydrate (P/C) ratio within the HFD on parameters of interest. Our data show that WPI has a specific effect on energy balance and gut microbiota, while increasing the P/C ratio within the HFD leads to dramatic alterations in energy balance, body composition, metabolic health and the composition of the gut microbiota.

## Materials and Methods

### Ethics Statement

All research involving mice was licensed under the Cruelty to Animal Act 1876 and received ethical approval from the University College Cork Animal Ethics Review Committee (#2011/005).

### Animals

Male 3–4 week old C57BL/6J mice (Harlan, Oxon, UK) were group housed either 5 per cage (Study 1) or 4 per cage (Study 2) in individually ventilated cages and acclimatised for four weeks in a light (06:00–18:00), temperature (21±1°C) and humidity (45–65%) controlled environment with free access to water and a low fat diet (LFD; 10% kJ fat and 20% kJ casein; #D12450, Research diets; New Brunswick, NJ, USA).

### Experimental protocol

Two studies were performed to assess how the WPI-derived energy content within a HFD (study 1) or LFD (study 2) impacts on energy balance-related parameters in mice over a 21 week (study 1) or 7 week period (study 2).

Study 1: Following the acclimatisation period, weight-matched dietary groups were maintained on the LFD or switched to either a HFD (45% kJ fat and 20% kJ casein; #D12451) or a HFD with WPI (Alacen^tm^ 895 NZMP, New Zealand) at an energy content of 20% kJ (HFD-20% WPI), 30% kJ (HFD-30% WPI) or 40% kJ (HFD-40% WPI) (Table S1) (n = 10) for a total of 21 weeks. Body weights were measured weekly. Energy intake in group housed mice was measured by weighing the food hopper each week until week 16. During weeks 17–20, energy intake and metabolic activity in individual mice was measured using TSE Phenomaster cages (TSE systems, Bad Homburg, Germany). Following this analysis and prior to re-housing the mice in home cages, faecal pellets were collected from individual mice for examination of microbial composition via pyrosequencing and subsequent bioinformatic analysis. At the end of the experimental period, mice were fasted for 6 hours and the body composition was measured using the Bruker minispec LF50H (Bruker optics, Ettlingen, Germany). Mice were then anesthetised using ketamine (65 mg/kg bodyweight) and xylazine (13 mg/kg bodyweight). Blood was collected from anesthetised mice into vacutinater EDTA tubes (BD, USA) and treated with Aprotinin (500,000 KIU/L final concentration; Sigma, Ireland) and Diprotin A (0.1 mM final concentration; Sigma, Ireland) to protect plasma peptides from proteolytic degradation. Plasma was isolated from blood by centrifugation at 2000 rpm at 4°C for 15 mins. Mice were sacrificed by cervical dislocation, and tissues of interest were dissected and snap frozen in liquid nitrogen (liver, adipose and stomach) or on dry ice (brain). Plasma and tissue samples were stored at −80°C until analysis.

Study 2: Weight matched mice were provided for 7 weeks with either the LFD or a LFD with WPI replacing the casein protein (LFD-WPI; 10% kJ fat and 20% kJ WPI) (n = 8). Body weights were measured weekly. Energy intake and metabolic activity in individual mice was measured during weeks 5 and 6 using the TSE Phenomaster system. After the analysis, mice were re-housed, as before, in the home cages and the experiment was terminated at the end of week 7.

### Analysis of metabolic parameters

The TSE Phenomaster cages comprised an open-circuit indirect calorimetry system with gas sensing units to measure oxygen consumption (ml/h/kg) (VO_2_) and CO_2_ production (ml/h/kg) (VCO_2_). The cages also contained high precision sensor associated-feeding baskets to accurately measure food intake (g), with a meal defined as intake over 0.01 g. A multi-dimensional infrared beam system allowed the measurement of locomotor activity, which was defined as the total number of infrared beam breaks in the X and Y axis. Mice were singly housed in TSE Phenomaster cages for a total of 3 days, with data collected during the final 24 hours, following a 2 day acclimatisation to the new cage environment. The acclimatisation period was established based on the data from our previous study [8]. Heat production (kcal/h/kg) in individual mice was calculated using the Weir equation (3.941×VO_2_ + 1.106×VCO_2_)[21]), and this was converted to kJ/h/kg using 1kcal  =  4.184 kJ. The respiratory exchange ratio (RER) was calculated by VCO_2_/VO_2_. Energy intake was calculated from food intake measurements using the energy content of the diets supplied by the manufacturer.

### Microbial DNA extraction, amplification and high throughput DNA sequencing

Total metagenomic DNA was extracted from individual faecal samples using QIamp DNA Stool Mini Kit (Qiagen, Hilden, Germany), after an additional bead-beating step. Bacterial composition was determined by sequencing of 16s rRNA amplicons (V4-V5 region; 408nt long) generated by a separate PCR reaction for each sample (in triplicate) using universal 16S primers, where, the forward primer (5′-AYTGGGYDTAAAGNG), with attached molecular identifier tags between 454 adapter sequence and target-specific primer sequence, and reverse primer V5 (5′-CCGTCAATTYYTTTRAGTTT) [22], were used along with Biomix Red (Bioline, London UK). The template DNA was amplified under the following PCR conditions for a total of 35 cycles: 94°C for 2 minutes and 1 minute respectively (initialization and denaturation), 56°C for 60 seconds (annealing) and 72°C for 60 seconds (elongation), proceeded by a final elongation stage of 2 minutes. Negative control reactions with PCR grade water in place of template DNA were used to confirm a lack of contamination. Amplicons were pooled and cleaned using the AMPure XP purification system (Beckman and Coulter, Takeley, UK) and DNA concentration was determined using the NANODROP 3300 Fluorospectrometer (Thermo Scientific, USA) coupled with the Quant-it™ Picogreen® dsDNA Assay Kit (Invitrogen, Paisley, UK). Equal volumes of each sample were then pooled together and underwent a final cleaning and quantification stage. Amplicons were sequenced in-house on a Roche GS FLX Titanium platform.

### Bioinformatics

Raw sequencing reads were ‘de-noised’ using traditional techniques implemented in the Ribosomal Database Project Pyrosequencing (RDP) Pipeline with ambiguous bases, non exact primer matches and reads shorter than 150 bp being excluded. Trimmed FASTA files were then BLASTed against a previously published 16S-specific database using default parameters. The resulting files were then parsed using the MEGAN software package, which assigns reads to the National Centre for Biotechnology Information (NCBI) taxonomies via the lowest common ancestor algorithm. Results were filtered prior to tree construction and summarization by the use of bit scores from within MEGAN where a cut-off bit score of 86 was employed [23], [24]. The QIIME software suite was employed to achieve clustering of sequence reads into operational taxonomic units (OTUs) [25]. Chimeric OTUs were removed using the ChimeraSlayer program [26] and phylogenetic trees constructed using the FastTreeMP tool [27]. Beta diversity values were calculated based on Bray Curtis, weighted and unweighted UniFrac distances, and the KING viewer was used to visualise resulting PCoA plots [28], [29]. Sequence reads were deposited in the European Nucleotide Archive (EHA) under the accession number PRJEB4636.

### Plasma analysis

Colorimetric assays were used to measure plasma levels of glucose (Calibochem, Darmstadt, Germany), triacylglycerol (TAG; Wako Chemicals, Richmond, VI, USA) and non-esterified fatty acids (NEFA; Abcam, Cambridge, UK). Commercially available ELISA kits were used to analyse plasma levels of insulin, leptin (Crystal Chem, Downers Grove, IL, USA), glucagon-like peptide 1 (GLP-1; Millipore, St. Charles, MO, USA) and corticosterone (Enzo Life sciences, Farmingdale, NY, USA). The homeostasis model assessment of insulin resistance (HOMA-IR) was determined using the formula: fasting plasma insulin (µU/ml) × fasting plasma glucose (mmol/L)/22.5 [30]. To measure plasma amino acid levels, samples were first deproteinised by mixing with equal volumes of 24% (w/v) tri-chloroacetic acid. The samples were then allowed to stand for 10 minutes before been centrifuged at 14400×g (Microcentaur, MSE, UK) for 10 minutes. Supernatants were mixed with 0.2 M sodium citrate buffer, pH 2.2, and the plasma concentration of amino acids were quantified using a Jeol JLC-500/V amino acid analyser (Jeol Ltd., Garden city, UK) fitted with a Jeol Na^+^ high performance cation exchange column.

### Liver TAG analysis

Total lipids from liver samples (approx. 50 mg) were extracted as described previously [8] using the Folch extraction method [31]. Briefly, total lipids were extracted using 2∶1 (v/v) chloroform: methanol solution, into which a 0.88% NaCl solution was added before centrifugation at 2000 rpm and 4°C for 30 mins. Aliquots of the organic phase were collected, dried and re-suspended in the LabAssay TAG reagent (Wako Chemicals, Richmond, VI, USA) to measure TAG levels using LabAssay TAG kit according to the manufacturer's protocol.

### Real-Time PCR analysis

Total RNA was isolated from tissues using RNeasy mini (liver and stomach) or RNeasy lipid mini (adipose and hypothalamus) kits (Qiagen, Hilden, Germany) according to manufacturers' instructions. RNA was treated with DNase (Qiagen, Hilden, Germany) during RNA isolation to eliminate any possible genomic DNA contamination. Reverse transcription of 1 µg of RNA was performed using 2.5 ng/µl random hexamer primers (Bioline, London, UK), 0.5 mM dNTP (Promega, Madison, VI, USA), 2 U/µl RNase inhibitor (Promega, Madison, VI, USA), and the Superscript II first stand system (Invitrogen, Carlsbad, CA, USA) according to manufactures' instructions. Gene expression was measured by the Roche Lightcycler 480 system (Rotkreuz, Switzerland) via amplification of 1 µl complementary DNA using the Lightcycler SYBR Green I Mastermix kit (Roche, Penzberg, Germany) and 2.5 µM gene specific primers (Eurofins MWG operon, Ebersberg, Germany) in a 10 µl total reaction volume. Primer sequences used are listed in Table S2. PCR conditions were; 10 mins at 95°C, followed by 50 cycles of 95°C for 10 s, 58–65°C for 5 s and 72°C for 15 s. Authenticity of PCR products was determined by melting curve analysis and by automated sequencing. Crossing point (Cp) of fluorescence signals were used to calculate target gene expression by 2^−ΔΔCp^, following normalisation against housekeeping gene according to ΔΔCp =  ΔCp target gene – ΔCp housekeeping gene. Housekeeping genes used were β-actin (liver, stomach and hypothalamus), YWHAZ (liver and hypothalamus), 18-S (adipose) and glyceraldehyde 3-phosphate dehydrogenase (GAPDH) (adipose and stomach). Relative gene expression is shown compared to the LFD group.

### Statistical analysis

Data are expressed as means ± standard error of the mean (SEM). Differences between experimental dietary groups were analysed by one-way or two-way ANOVA followed by pairwise comparisons using *tukey* or *bonferroni* post hoc tests, respectively. Body weight trajectories were analysed by two-way repeated measures ANOVA with *bonferroni* post hoc tests. Non-parametric data was compared by Kruskal-Wallis ANOVA followed by *Dunn*'*s* pairwise comparisons. Significance levels were set at *P*≤0.05, and statistical analysis was performed using Graphpad prism (ver. 5.04; San Diego, CA, USA) and Minitab (ver.15; State College, PA, USA).

## Results

### WPI inclusion or increasing the P/C ratio within a HFD alters body composition and plasma amino acids

**Figure 1:**
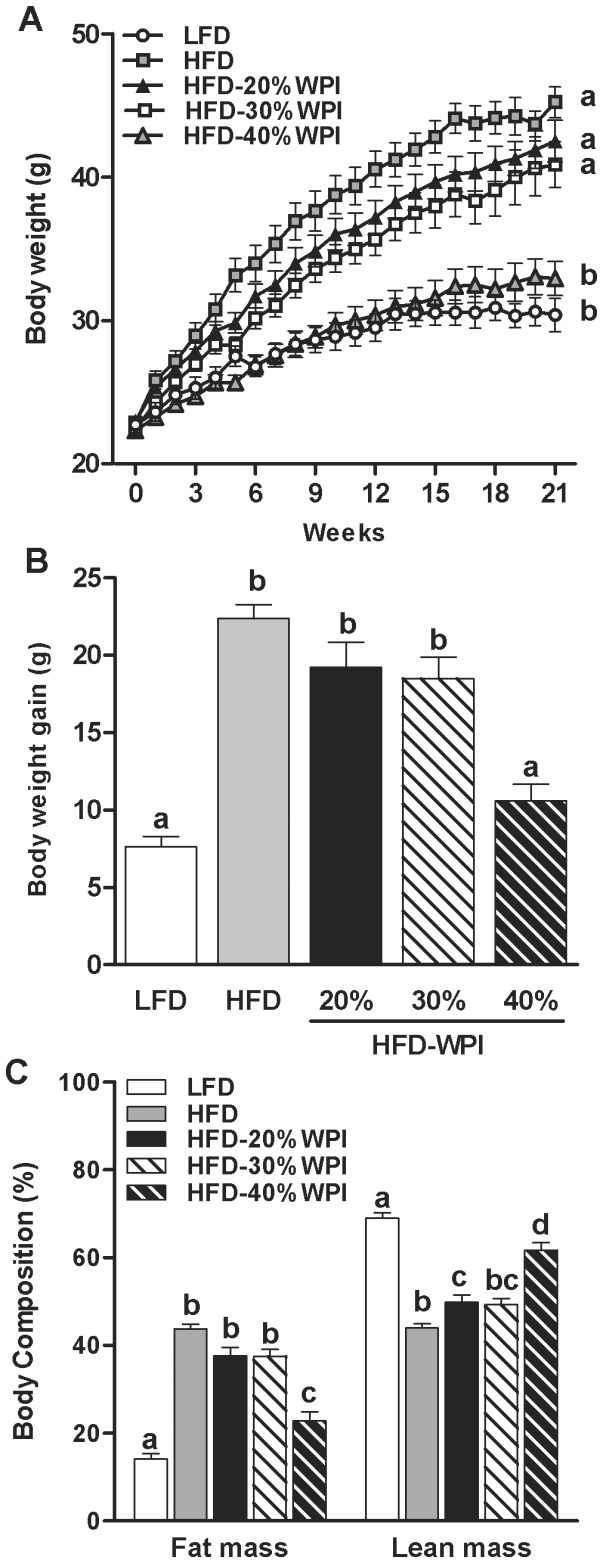
Impact of whey protein isolate and protein to carbohydrate ratio on body weight and composition. (A) shows the body weight trajectories of mice during 21 weeks of dietary treatment with a 10% kJ low fat diet (LFD), 45% kJ high fat diet (HFD) or a HFD with 20, 30 or 40% kJ whey protein isolate (WPI). Body weight gain (B) and body composition (C) of mice after 21 weeks on experimental diets are also shown. Data represent mean values ± S.E.M. (n = 10 per group). Groups that do not share a common letter are significantly different at *P<*0.05.

Fig. 1A–B demonstrates that body weight gain of HFD-20%WPI fed mice was similar to HFD controls. However, intake of WPI was seen to have a specific effect on body composition, with HFD-20% WPI fed mice having an increased lean mass (%) (*P<*0.05), and a trend towards a reduction in fat mass (%) (*P = *0.08) compared to the HFD control group. Increasing the WPI derived energy content in the HFD to 40% and proportionally reducing the carbohydrate energy content led to a significant reduction in body weight gain compared to all other HFD-WPI groups (*P<*0.001), with observed values similar to that seen for the LFD group. This was accompanied by significantly reduced body fat mass and increased lean mass levels in the HFD-40% WPI fed mice compared to HFD control and other WPI diets groups (*P<*0.001), while the body composition of HFD-30% WPI group did not differ from that of the HFD-20% WPI group (Fig. 1C).

Comparison of the plasma amino acid profiles including those that could influence lean and fat mass, revealed an impact of WPI and the P/C ratio (Table 1). WPI specific effects were observed on glutamic acid, aspartic acid and glycine, which either decreased (glutamic acid and aspartic acid) or increased (glycine) compared to HFD fed mice (*P<*0.05) (Table 1). Changes in macronutrient ratio in HFD-40%WPI, decreased plasma histidine, phenylalanine, serine and threonine levels compared to the lowest P/C ratio (20% WPI) (*P<*0.01) (Table 1).

**Table 1:** Plasma amino acid levels (µmol/L) in mice fed a 45%kJ high fat diet (HFD) or HFD with 20%, 30% or 40% kJ whey protein isolate (WPI) for 21 weeks^1^.

	HFD	20% WPI	30% WPI	40% WPI	P value
Alanine	170.91±8.82^a^	163.83±11.59^ab^	137.44±9.72^ab^	127.15±8.46^b^	<.05
Arginine	41.74±4.82	38.41±7.16	56.39±7.60	39.08±4.34	NS
Aspartic acid	7.47±1.25^a^	3.63±0.51^b^	3.49±0.51^b^	4.85±1.02^ab^	<.05
Cyteine	11.95±1.34	8.87±1.37	8.93±1.33	7.86±1.72	NS
Glutamic acid	90.76±3.42^a^	74.74±1.89^b^	75.25±1.86^b^	71.25±2.69^b^	<.001
Glycine	106.22±4.28^a^	131.43±3.57^b^	116.45±4.52^ab^	131.92±2.69^b^	<.001
Histidine	75.43±2.12^a^	74.93±2.98^a^	68.21±3.35^ab^	64.21±2.51^b^	<.05
Isoleucine	213.81±8.87	207.87±9.09	195.27±5.74	186.78±10.17	NS
Leucine	91.86±9.66	81.36±6.65	66.93±6.69	69.71±10.21	NS
Lysine	113.81±5.88	108.18±8.30	102.57±8.39	88.29±4.07	NS
Methionine	23.94±1.77	23.65±0.75	21.05±0.97	20.40±1.08	NS
Phenylalanine	46.92±1.70^a^	41.13±1.13^ab^	38.23±1.52^bc^	33.30±2.35^c^	<.001
Proline	118.26±10.14	127.63±9.63	106.04±11.02	104.39±11.93	NS
Serine	68.18±5.69^ab^	74.98±2.29^a^	62.42±2.57^ab^	55.73±2.12^b^	<.01
Threonine	92.71±2.93^a^	87.63±2.49^a^	78.29±2.95^ab^	69.28±3.96^b^	<.01
Tyrosine	40.29±2.47^a^	36.37±1.12^ab^	33.76±1.54^ab^	29.63±2.77^b^	<.01
Valine	154.51±8.65^a^	130.59±5.99^ab^	118.40±6.69^b^	113.16±9.49^b^	<.01

1 Data are means ± SEM (n = 7–10). In each row values without a common letter significantly differ, *P*<0.05;NS, non-significant.

### WPI-enriched HFD normalised energy intake, while increasing the P/C ratio accentuated metabolism

**Figure 2:**
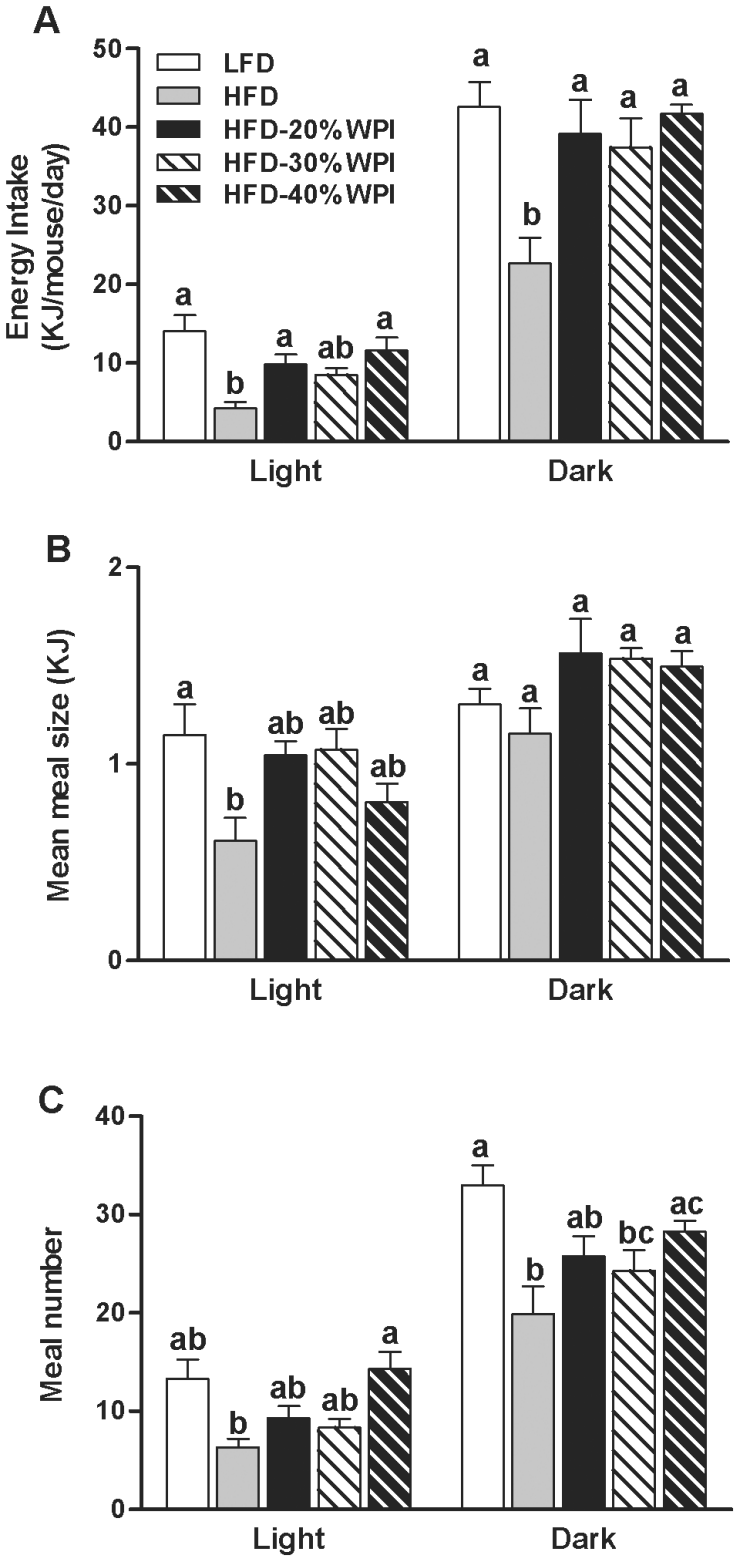
Impact of whey protein isolate and protein to carbohydrate ratio on energy intake. Energy intake (A) and feeding behaviour (meal size & meal number) (B–C) was measured using TSE Phenomaster cages at 17–20 weeks for mice on either a 10% kJ low fat diet (LFD), 45% kJ high fat diet (HFD) or a HFD with 20, 30 or 40% kJ whey protein isolate (WPI). Experimental data collected from individual mice at 9 minute intervals over a 24 hour period are shown as mean values ± SEM (n = 8–10 per group) for light and dark phases. In light and dark phase, groups that do not share a common letter are significantly different at *P<*0.05.

The cumulative energy intake (MJ) for the dietary groups (2 cages/group, all with n = 5 mice), measured over the first 16 weeks did not differ between LFD, HFD and HFD-20% WPI groups (24.73±2.70 vs. 27.69±1.54 vs. 29.40±0.31, respectively). In contrast, data gathered by individually housing the mice in TSE Phenomaster cages in weeks 17–20 demonstrated that the energy intake of the HFD-WPI groups was greater than that of the HFD control group during both the light and dark phases (*P<*0.05), while being similar to that of the LFD group (Fig. 2A). Increasing the P/C ratio had no significant effect on cumulative energy intake (MJ) in HFD fed mice up to week 16 (20%WPI, 29.40±0.31 vs. 30%WPI, 30.01±0.62 vs. 40%WPI, 26.71±0.15). Energy intake measurements from TSE Phenomaster cages corroborated this data (Fig 2A). There was also no significant effect on meal number or meal size of altering the P/C ratio (i.e. between WPI groups; Fig 2B–C).

**Figure 3:**
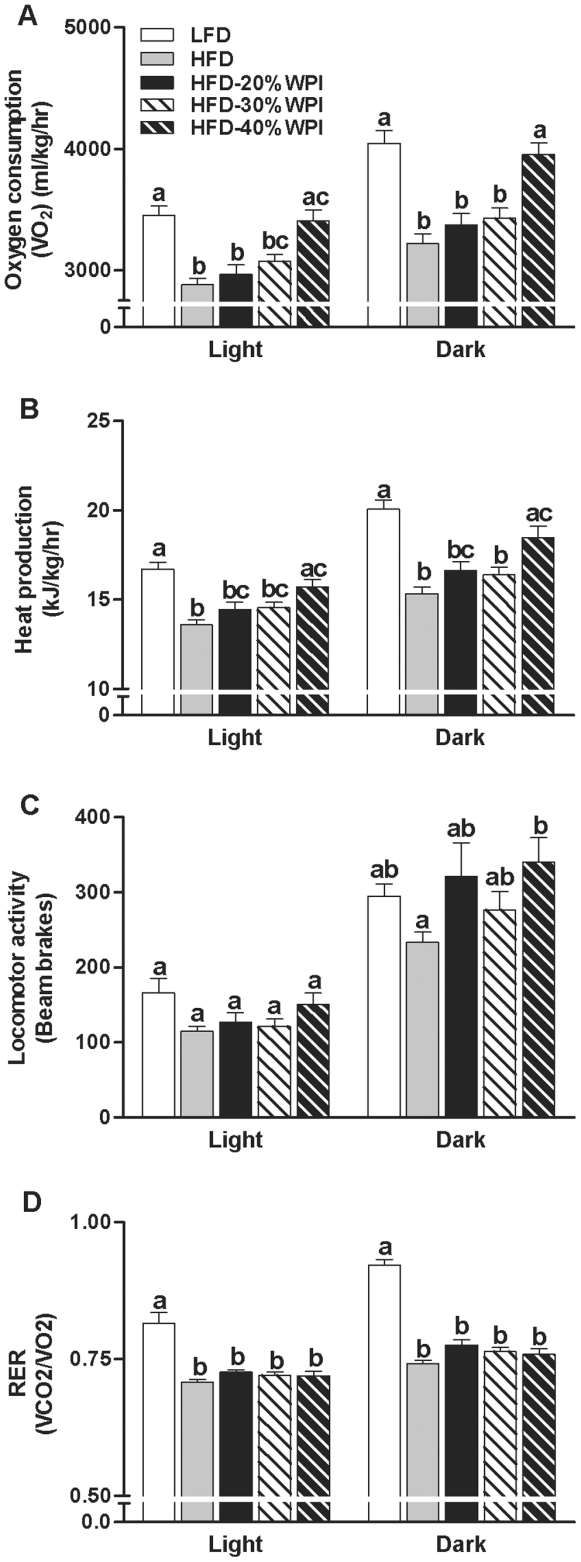
Impact of whey protein isolate and protein to carbohydrate ratio on metabolic activity. Metabolic activity was measured using TSE Phenomaster cages at 17–20 weeks for mice on either a 10% kJ low fat diet (LFD), 45% kJ high fat diet (HFD) or a HFD with 20, 30 or 40% kJ whey protein isolate (WPI). Experimental data for (A) oxygen consumption (VO_2_), (B) heat production, (C) locomotor activity and (D) respiratory exchange ratio (RER), collected from individual mice at 9 minute intervals over a 24 hour period, are shown as mean values ± SEM (n = 8–10 per group) for light and dark phases. In light and dark phase, groups that do not share a common letter are significantly different at *P<*0.05.

The HFD-20% WPI diet had no impact on VO_2_, heat production, locomotor activity or respiratory exchange ratio (RER) when compared to HFD fed mice (Fig. 3A–D). Increasing the P/C ratio was found to impact on energy expenditure with HFD-40% WPI fed mice having significantly increased levels of dark phase VO_2_ compared to HFD-20 and 30% WPI fed mice (*P<*0.001) (Fig 3A). A similar change in heat production was observed between the groups, albeit data was only significant between HFD-40% and HFD-30% WPI groups (*P<*0.05) (Fig 3B). There was no effect of WPI or P/C ratio on locomotor activity (Fig 3C). RER values of all HFD groups were lower than the LFD group in both the light and dark phases, consistent with increased fat metabolism (*P<*0.001) (Fig 3D).

Investigation of the above parameters in mice fed a LFD with WPI or casein for 7 weeks (study 2) revealed that WPI does not influence body weight, energy intake, VO_2_, locomotor activity or RER in a low fat background (Figure S1A–D).

### Increasing the protein to carbohydrate ratio attenuated adverse metabolic impact of HFD

**Figure 4:**
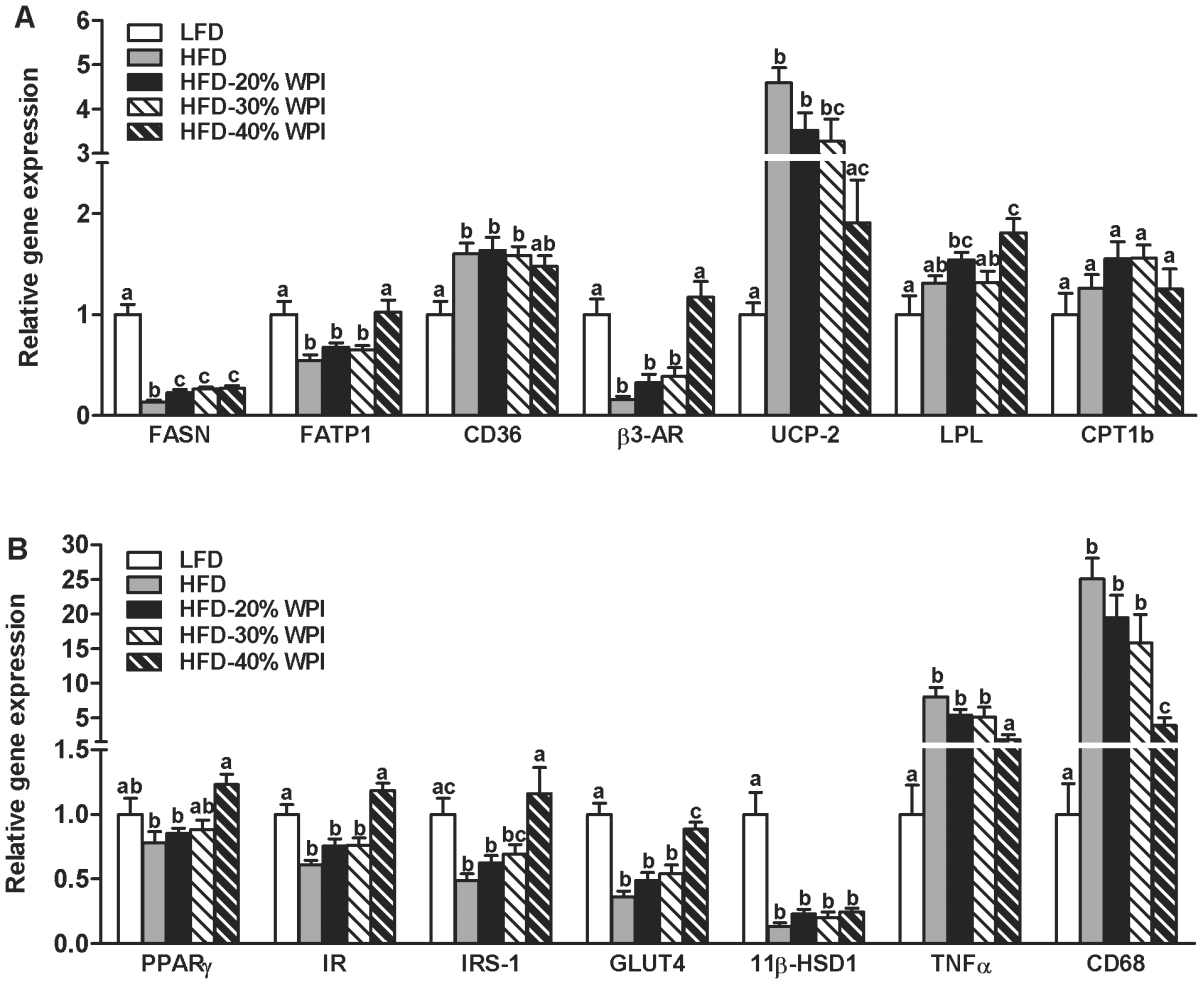
Impact of whey protein isolate and protein to carbohydrate ratio on adipose cellular activity. Epididymal adipose tissue gene expression was investigated in mice after 21 weeks on a 10% kJ low fat diet (LFD), 45% kJ high fat diet (HFD), or HFD with 20, 30 or 40% kJ whey protein isolate (WPI). Relative mRNA expression is shown for (A) fatty acid synthase (FASN), fatty acid transporter 1 (FATP1), cluster of differentiation 36 (CD36), beta-3 adrenergenic receptor (β3-AR), uncoupling protein 2 (UCP-2), lipoprotein lipase (LPL) and carnitine palmitolytransferase 1b (CPT1b), and (B) for peroxisome proliferator activated receptor gamma (PPARγ), insulin receptor (IR), insulin receptor substrate 1 (IRS-1), glucose transporter 4 (GLUT4), 11β-hydroxysteroid dehydrogenase type 1 (11β-HSD1), tumour necrosis factor alpha (TNF-α) and cluster of differentiation 68 (CD68). Data represent mean values ± SEM (n = 9–10 per group). Gene expression is shown relative to the LFD control group set at 1.00. Groups that do not share a common letter are significantly different at *P<*0.05.

Specific effects of WPI and the P/C ratio were observed on lipid metabolism-related gene expression and on tissue lipid deposition. Firstly, the decrease in epididymal adipose tissue fatty acid synthase (FASN) mRNA expression with HFD feeding, was somewhat attenuated by WPI challenge (*P<*0.05), with no added benefit of increasing the P/C ratio on expression of this gene (Fig 4A). Notably, the epididymal mRNA expression of a number of other genes were altered by the P/C ratio, specifically, fatty acid transporter 1 (FATP1), beta-3 adrenergic receptor (β3-AR), peroxisome proliferator-activated receptor gamma (PPARγ), uncoupling protein 2 (UCP-2) and lipoprotein lipase (LPL) (*P*≤0.05) (Fig 4). In the liver, WPI specifically reduced TAG levels (Table 2) and the mRNA expression of fatty acid binding protein 1 (FABP1) compared to HFD fed mice (Table 3). The highest P/C ratio (40% WPI) significantly decreased mRNA levels of cluster of differentiation 36 (CD36) and PPARγ (*P<*0.05) (Table 3), dramatically reduced liver TAG levels compared to 20/30% WPI fed mice (Table 2), and normalised the elevated plasma levels of TAG and NEFA observed with HFD feeding (*P*≤0.05) (Table 2). Finally, the mRNA level of lipid metabolism-related carnitine palmitoyltransferase 1a-c (CPT1a-c), fatty acid transport protein 5 (FATP5) and PPARα, in tissues of interest, was not influenced by the dietary challenges (Tables 3–4).

**Table 2:** Tissue lipid parameters and plasma levels of hormones and metabolites in mice fed a 10%kJ low fat diet (LFD), 45%kJ high fat diet (HFD) or HFD with 20%, 30% or 40% kJ whey protein isolate (WPI) for 21 weeks^1^.

	LFD	HFD	20% WPI	30% WPI	40%WPI	P value
Liver TAG (mg/g tissue)	71.47±9.17^a^	139.03±6.92^b^	104.94±5.88^c^	107.21±8.83^c^	70.82±7.64^a^	<.001
Leptin (ng/ml)	4.41±0.75^a^	84.69±3.17^b^	53.78±5.67^c^	51.12±4.69^c^	15.01±4.07^a^	<.001
TAG (mg/dl)	39.85±2.91^a^	56.90±6.31^b^	45.15±4.32^ab^	52.36±3.91^ab^	40.19±3.96^a^	<.05
NEFA (mmol/L)	0.37±0.09^a^	0.67±0.07^b^	0.64±0.05^b^	0.63±0.06^b^	0.36±0.05^a^	<.01
Corticosterone (ng/ml)	150.0±21.7^a^	319.0±48.6^b^	286.8±31.9^ab^	334.1±36.7^b^	277.1±35.5^ab^	<.01
GLP-1 (pM)	25.03±2.47	33.15±2.83	28.07±2.3	27.88±1.98	24.03±2.25	NS
Glucose (mmol/L)	9.14±1.24^a^	15.68±0.83^b^	13.92±0.95^b^	14.08±1.05^b^	9.25±0.77^a^	<.001
Insulin (ng/ml)	0.29±0.05	0.55±0.08	0.40±0.10	0.37±0.04	0.31±0.06	NS
HOMA-IR	2.48±0.67^a^	9.79±1.07^b^	6.81±2.58^ab^	6.20±0.95^ab^	3.04±0.64^a^	<.01

1 Data are means ± SEM (n = 5–10). In each row results without a common letter significantly differ, *P*≤0.05 NS, non-significant. TAG, triacylglycerol. NEFA, non-esterified fatty acids. GLP-1, glucagon-like peptide 1. HOMA-IR, homeostasis model assessment of insulin resistance.

**Table 3:** Relative hepatic gene expression in mice fed a 10%kJ low fat diet (LFD), 45%kJ high fat diet (HFD) or HFD with 20%, 30% or 40% kJ whey protein isolate (WPI) for 21 weeks^1^.

	LFD	HFD	20% WPI	30% WPI	40% WPI	P value
CD36	1.00±0.20^a^	2.85±0.34^b^	2.98±0.45^b^	2.17±0.25^ab^	1.50±0.25^a^	<.05
PPARγ	1.00±0.12^a^	3.00±0.57^b^	2.30±0.49^b^	1.88±0.46^b^	0.85±0.13^a^	<.001
FABP1	1.00±0.09^ab^	1.27±0.10^b^	0.86±0.06^ac^	1.27±0.17^bc^	0.71±0.09^a^	<.001
IRS-1	1.00±0.15^a^	0.58±0.04^b^	0.69±0.05^ab^	0.68±0.06^ab^	0.79±0.06^ab^	<.01
CPT1a	1.00±0.08	0.85±0.08	0.97±0.06	0.90±0.13	0.86±0.10	NS
GLUT2	1.00±0.09	0.76±0.04	1.02±0.08	0.97±0.09	0.89±0.05	NS
FATP5	1.00±0.21	0.89±0.07	0.88±0.07	1.16±0.07	1.24±0.12	NS
FASN	1.00±0.36	0.46±0.14	0.54±0.19	0.40±0.06	0.32±0.04	NS
PPARα	1.00±0.04	1.13±0.05	1.21±0.05	1.17±0.10	1.28±0.11	NS
TNFα	1.00±0.26	0.84±0.15	1.12±0.18	0.80±0.09	0.71±0.10	NS

1 Data are means ± SEM (n = 7–10). In each row results without a common letter significantly differ, *P<*0.05; NS, non-significant. Gene expression shown relative to the LFD control group set at 1.00.CD36, cluster of differentiation 36; PPARγ, peroxisome proliferator activated receptor gamma; FABP1, Fatty acid binding protein 1; IRS-1, Insulin receptor substrate 1; CPT1a, carnitine palmitoyltransferase 1a; GLUT2, Glucose transporter 2; FATP5, Fatty acid transporter 5; FASN, Fatty acid synthase; TNF-α, Tumour necrosis factor alpha.

**Table 4:** Relative hypothalamic gene expression in mice fed a 10%kJ low fat diet (LFD), 45%kJ high fat diet (HFD) or HFD with 20%, 30% or 40% kJ whey protein isolate (WPI) for 21 weeks^1^.

	LFD	HFD	20% WPI	30% WPI	40% WPI	P value
IR	1.00±0.03^ab^	0.97±0.07^a^	1.22±0.04^bc^	1.37±0.04^cd^	1.55±0.08^d^	<.05
GCCR	1.00±0.09^a^	1.40±0.10^b^	1.34±0.07^ab^	1.30±0.08^ab^	1.42±0.09^b^	<.05
TNFα	1.00±0.05^a^	1.76±0.09^b^	1.57±0.13^b^	1.47±0.14^ab^	1.40±0.25^ab^	<.01
POMC	1.00±0.08	0.99±0.06	1.01±0.06	0.98±0.06	1.00±0.08	NS
NPY	1.00±0.04	1.02±0.09	0.90±0.06	0.88±0.05	0.92±0.08	NS
ObR	1.00±0.11	0.92±0.05	0.99±0.05	1.07±0.07	0.95±0.05	NS
GHS-R	1.00±0.07	0.98±0.06	0.98±0.08	1.02±0.11	1.04±0.09	NS
PPARγ	1.00±0.36	1.07±0.04	0.95±0.05	0.99±0.05	0.98±0.07	NS
CPT1c	1.00±0.09	0.92±0.04	0.91±0.01	0.97±0.06	0.86±0.04	NS
IRS-1	1.00±0.04	0.99±0.04	0.97±0.02	1.06±0.08	1.00±0.06	NS

1 Data are means ± SEM (n = 5–10). In each row values without a common letter significantly differ, *P*<0.05; NS, non-significant. Gene expression shown relative to the LFD control group set at 1.00. IR, Insulin receptor; GCCR, Glucorticoid receptor; TNF-α, Tumour necrosis factor alpha; POMC, Pro-opiomelancortin; NPY, Neuropeptide Y; ObR, Leptin receptor; GHS-R, Growth hormone secretatgogue receptor; PPARγ, peroxisome proliferator activated receptor gamma; CPT1c, carnitine palmitoyltransferase 1c; IRS-1, Insulin receptor substrate 1.

Increasing the P/C ratio reduced plasma glucose levels, particularly in the 40% WPI group (*P<*0.05) (Table 2). In parallel HOMA-IR values were also reduced (*P<*0.05), but the change in plasma insulin concentration did not reach statistical significance (Table 2). At a cellular level, the highest P/C ratio normalised the HFD-induced reduction in adipose expression of insulin receptor (IR) and insulin receptor substrate 1 (IRS-1), and partially prevented the HFD-induced reduction in glucose transporter 4 (GLUT4) (Fig. 4B) (*P<*0.001). In the hypothalamus, WPI specifically increased IR mRNA expression (*P<*0.05), as did P/C ratio, with the highest P/C ratio having the greatest impact (Table 4). Epididymal adipose tissue mRNA expression of inflammatory markers, namely tumour necrosis factor (TNF)-α and cluster of differentiation (CD) 68 only responded to the highest P/C ratio, which significantly reduced the expression of both in a HF background (Fig. 4B) (*P<*0.001). In the hypothalamus, whilst TNFα mRNA was elevated by HFD feeding, neither WPI nor the P/C ratio influenced its levels, although there was a trend towards a decrease for the highest P/C ratio (40% WPI) (Table 4). None of the dietary challenges influenced hepatic glucose transporter 2 (GLUT2), IRS-1 and TNF-α mRNA expression (Table 3) or hypothalamic IRS-1 mRNA expression (Table 4).

The increased plasma leptin concentration in response to the HFD was significantly blunted by WPI intake with dramatic reductions seen at the highest P/C ratio (*P<*0.001) (Table 2). Yet, the hypothalamic expression of genes known to be responsive to plasma leptin levels were unaffected, specifically mRNA levels of leptin receptor (ObR), pro-opiomelanocortin (POMC), neuropeptide Y (NPY) and growth hormone secretagogue receptor (GHS-R) (Table 4). In addition, gastric mRNA expression for the orexigenic hormone ghrelin was not found to significantly differ between all dietary treatment groups (1.00±0.37, LFD vs. 1.11±0.40, HFD vs. 0.73±0.35, 20%-WPI vs. 0.29±0.28, 30%-WPI vs. 1.07±0.45, 40%-WPI). Plasma corticosterone levels were also elevated with HF feeding, but were not influenced by protein source (WPI or casein) or P/C ratio (Table 2). Similarly, there was no effect of WPI on the HFD-associated suppression of adipose 11β-hydroxysteroid dehydrogenase type 1 (11βHSD1) (Fig 4B), or the HFD-induced increase in glucocorticoid receptor (GCCR) in the hypothalamus (Table 4).

### WPI inclusion or increasing the P/C ratio within a HFD altered the composition of gut microbiota

**Figure 5:**
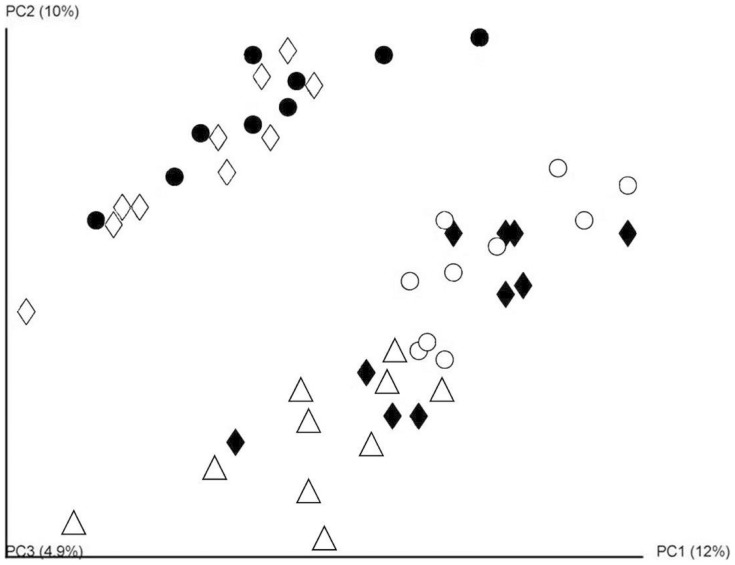
Impact of whey protein isolate and protein to carbohydrate ratio on the gut microbiota composition. Principal Coordinate analysis (PCoA) of unweighted Unifrac distances of the 16srRNA sequences, demonstrating where sequences cluster according to diet group. Data were generated from analysis of faecal samples collected from mice on 10% kJ low fat diet (LFD, Δ) or 45% kJ high fat diet (HFD, ♦) or a HFD with 20% kJ whey protein isolate (HFD-20% WPI, ○), 30% kJ WPI (HFD-30% WPI, ◊) or 40% kJ WPI (HFD-40% WPI, •)(n = 10).

A total of 251,395 V4–V5 16s rRNA sequence reads were generated which corresponded to an average of 50,279 reads per diet group or 5,130 reads per animal. α-diversity values were calculated for biodiversity (Shannon index), species richness (Chao1) and the number of species relative to the abundance in the sample (Simpson diversity index). When α-diversity values were compared by diet group, the only difference observed was a significantly higher microbial richness (Chao1) within the HFD microbiota compared to the HFD-30% WPI (*P = *0.028). Principal coordinate analysis (based on unweighted UniFrac distances) (Fig. 5) of the sequence data highlighted a clustering of the LFD, HFD and HFD-20% WPI group microbial populations, while HFD-30% and 40% WPI groups clustered in close proximity to each other and distinctly from the LFD, HFD and HFD-20% WPI group clusters. Indeed, the LFD diet with casein as a protein source clustered most closely with the HFD containing the casein protein.

Phylogenetic analysis revealed several significant microbial population shifts between the HFD control and WPI groups (Table 5). At the family level, all WPI diet groups had significantly increased proportions of *Lactobacillaceae* and significantly decreased proportions of *Clostridiaceae* compared to the HFD control group. *Bifidobacteriaceae* populations were increased in both the HFD-20% WPI and HFD-30% WPI diet groups compared to the HFD control, while in contrast they were significantly lower in the HFD-40% WPI group compared to the HFD control. The aforementioned patterns were also observed with respect to the corresponding genera (*Lactobacillus, Clostridium* and *Bifidobacterium* respectively) (Table 5). Also at genus level, proportions of *Rikenella* were significantly higher in the HFD-40% WPI group compared to HFD-20/30% WPI groups, while proportions of *Peptostreptococcus* were significantly higher in the HFD-40% WPI group than in any other diet group (Table 5). Specific comparison of the microbiota of the HFD-20% WPI and HFD control was deemed particularly important given that the changes occurring here reflected changes resulting specifically from the presence of whey protein, rather than casein, in the diet, and not simply a change in the P/C ratio in the diet. In addition to the changes in the *Lactobacillus*, *Clostridium* and *Bifidobacterium* populations (and associated families) referred to above, it was also noted that proportions of *Desulfovibrio* and *Mucisprillum* (genus) were increased in the HFD-20% WPI relative to HFD control animals.

**Table 5:** Gut microbiota composition as % of reads in mice fed a 45%kJ high fat diet (HFD) or HFD with 20%, 30% or 40% kJ whey protein isolate (WPI) for 21 weeks^1^.

	HFD	20% WPI	30% WPI	40% WPI
**Phylum**				
*Proteobacteria*	0.36^a^	0.63^b^	0.34^ab^	0.32^a^
*Actinobacteria*	0.63^a^	1.82^b^	3.79^b^	0.36^c^
*Deferribacteres*	0.57^a^	1.61^b^	1.56^ab^	2.03^b^
**Family**				
*Desulfovibrionaceae*	0.12^a^	0.31^b^	0.21^ab^	0.23^ab^
*Rikenellaceae*	6.71^ab^	7.54^b^	3.9^a^	6.4^ab^
*Bacteroidaceae*	0.44^a^	0.42^a^	0.16^b^	0.21^b^
*Lactobacillaceae*	0.21^a^	3.03^b^	4.6^b^	2.14^b^
*Bifidobacteriaceae*	0.43^a^	1.71^b^	3.66^b^	0.22^c^
*Deferribacteraceae*	0.57^a^	1.59^b^	1.32^ab^	2.03^ab^
*Peptostreptococcaceae*	0.62^a^	1.79^a^	1.54^a^	8.01^b^
*Succinivibrionaceae*	0.13^a^	0.15^a^	0^b^	0^b^
*Clostridiaceae*	1.31^a^	0^b^	0^b^	0^b^
*Veillonellaceae*	0.02^a^	0.12^b^	0^a^	0^a^
**Genus**				
*Anaerobiospirillum*	0.13^a^	0.15^a^	0^b^	0^b^
*Desulfovibrio*	0.07^a^	0.22^b^	0.17^ab^	0.15^ab^
*Alistipes*	4.33^ab^	4.41^a^	2.24^b^	3.76^ab^
*Rikenella*	1.04^ab^	0.49^b^	0.68^b^	1.08^b^
*Bacteroides*	0.44^a^	0.4^a^	0.16^b^	0.21^ab^
*Oscillibacter*	0.24^a^	0.67^ab^	0.42^ab^	0.52^b^
*Lactobacillus*	0.2^a^	3.03^b^	4.6^b^	2.39^b^
*Bifidobacterium*	0.43^a^	1.71^b^	3.66^b^	0.22^c^
*Mucispirillum*	0.57^a^	1.61^b^	1.56^ab^	1.92^ab^
*Coprococcus*	0.11^ab^	0.23^b^	0.06^a^	0.06^ab^
*Turicibacter*	0.56^a^	0.35^a^	0.15^ab^	0^b^
*Clostridium*	1.3^a^	0^b^	0^b^	0^b^
*Peptostreptococus*	0.1^a^	0.14^a^	0.12^a^	0.78^b^

1 Data are means ± SEM (n = 10). Statistically significant differences generated using the Kruskal-Wallis algorithm. In each row values without a common letter significantly differ, *P*≤0.05.

## Discussion

The key findings of this study are that WPI has a specific effect on HFD-induced energy intake, metabolic health and gut microbiota composition. Additionally, with the exception of energy intake, increasing the P/C ratio, by increasing WPI dietary content, was seen to dramatically alter the above parameters.

### Energy intake

Cumulative energy intake measured up to week 16 did not significantly differ between LFD and HFD fed mice. While this is consistent with data reported elsewhere [20], HF feeding has also been shown to increase or decrease energy intake in rodents [32], [33]. Differences between data reported may relate to variances in diet composition including fat source/composition, or it may be due to differences in the palatability of the LFD used as the control [33], [34].

In this study, there was a discrepancy in energy intake in HFD fed mice depending on the housing environment (single or group housed). In contrast to the group house environment up to week 16, HFD fed mice when individually housed in metabolic cages during weeks 17–20 showed a hypophagic response compared to LFD fed mice. It is possible that these differences may be related to the accuracy of the method used to measure food intake in group versus single housed mice, although if this was an error due to methodology, then it would likely to have influenced all dietary groups equally and not just the HFD group. Alternatively, the different behavioural responses could be result of social isolation, which has been shown to decrease energy intake and elevate plasma corticosterone levels [35], [36]. However, socially isolated mice adapt to the new environment and consume similar amounts of food as pre-adapted singularly housed mice 6 hr post-novelty stress, but interestingly 24hr later, their food intake reduces significantly once again in the new environment, suggesting that stress of social isolation could extend up to 24 h [36]. We showed that group housed mice on a LFD when placed in isolation adapt to the new environment and continue to consume similar amounts of food by day 2 and 3 in the new location [8] and consequently used the day 3 time point to measure energy intake in this study. Rodents on a HFD have been shown to display increased anxiety [37], and have an over-active hypothalamic-pituitary-adrenal axis [38], [39] resulting in elevated plasma corticosterone levels, as demonstrated here. This could explain why HFD fed mice are more susceptible to stress stimuli with more pronounced reductions in energy intake compared LFD fed counterparts subjected to the same stress levels [40]–[42]. Consequently, in a HFD background, it's possible that social isolation-induced stress responses could have had a greater impact on energy intake with effects lasting up to the 3 day housing period as used in this study. Given the finding that whey proteins such as lactoferrin and α-lactalbumin and native whey protein itself reduce stress [43]–[47], it is possible that replacing the casein protein with an equivalent WPI content caused normalisation of energy intake in HFD-fed mice by affecting a specific feeding behaviour related mechanism(s), with increasing the WPI protein-derived bioactives having no further effect. This WPI effect on energy intake appeared be specific to the HFD-induced neuroendocrine state because mice on the LFD with WPI showed similar energy intake to casein diet-fed controls. Since neither WPI nor increasing the P/C ratio influenced plasma corticosterone levels, adipose expression of 11β-HSD1 or hypothalamic expression of GCCR in HFD fed mice, it is possible that WPI may have affected other central mechanisms mediating stress responses not investigated in this study [48]–[50] either independently or in combination with key mechanisms regulating energy balance. Given that leptin decreases meal size and number [51]–[53], and WPI reduced the HFD-induced increase in plasma leptin levels, it's possible that WPI-derived bioactives could have specifically influenced circadian rhythm of leptin production and/or action within the neuroendocrine state of HFD-fed mice in a socially isolated environment. Additionally, the reduction in plasma amino acids associated with WPI intake (see below), could also have acted as a possible central trigger to increase energy intake in WPI groups compared to HFD control in the single house environment.

HFD feeding has been shown to cause a gain of weight in rats up to the duration of a test period lasting 76 weeks, with animal's body weight gain responding to changes to the dietary fat content introduced at various time points [54]. Lin *et al.,* [32] demonstrated that mice on a HFD for 19 weeks are responsive to intracerebroventricular administration of leptin. These data suggest that energy balance related mechanisms are able to respond to energetic challenges even after prolonged high fat intake. High protein intake within a HFD suppresses energy intake [55], [56] albeit not consistently [4], [57], and in our study, the P/C ratio did not alter energy intake in either housing environment. This could be a result of the quantity or composition of the macronutrient used in the test diets. Indeed, data from human trials showed that increasing protein dietary content (10/15% to 30%) only decreased energy intake when the carbohydrate content was kept constant [58], [59]. This further highlights the importance of designing appropriate experimental diets with the correct macronutrient composition for uncovering the energy balance related impact of the dietary component under investigation.

### Metabolic health

Replacement of the casein protein with an equivalent energy content of WPI (i.e. 20%) did not specifically alter metabolic activity, heat production or locomotor activity in HFD or LFD fed mice. In contrast, Acheson *et al.,* [60] showed that whey has a greater thermic effect than casein or soy in humans. These differences in data may be related to the fact that the latter study only investigated an acute post-prandial response to a defined test meal, or it may relate to how different species (humans versus mice) digest and metabolise dietary proteins. Shetzer *et al,* found that mice consuming a HFD and WPI-supplemented drinking water have enhanced oxygen consumption compared to mice drinking unsupplemented water [4]. In this instance, the increased metabolic activity may have arisen due to the increased protein intake (proteins from diet and from WPI supplemented water). In fact, this corroborates with the data presented here, which show that increasing the P/C ratio resulted in increased energy expenditure (VO_2_ and heat production) and dark phase locomotors activity, resulting presumably from the increased catabolism of ingested dietary protein, coupled with thermic effects of WPI compared to casein [60] and/or due to increased deposition of lean mass with WPI content [61]–[63]. Interestingly, Zhang *et al.* [64] showed that HFD fed mice on leucine-supplemented drinking water have reduced fasting plasma levels of aspartic acid, glutamic acid, and phenylalanine, as well as increased VO_2_ and reduced adiposity compared to HFD controls. Given the influence of leucine on WPI-induced muscle hypertrophy [65], [66] and its unique ability to regulate the translation of protein synthesis [67], it is possible that the elevated leucine content found normally in the WPI diets may have enhanced muscle protein synthesis by directing other amino acids towards protein synthesis and/or catabolism [68], [69], with the required energy been derived possibly from fat catabolism [70]. Consistent with the latter suggestion, we found an increased lean mass and a trend towards a reduction in fat mass with decreased plasma levels of several amino acids, but not leucine, when the casein protein in a HFD was replaced with WPI or when the P/C ratio in the HFD was increased.

WPI intake appeared to cause a trend towards a reduction in fat mass, and in the liver this manifested as a WPI specific reduction in TAG levels, which was accompanied by the suppression of FABP1 mRNA expression, similar to previous findings [4], [6], [71], [72]. In the epididymal adipose tissue, WPI prevented the HFD-induced FASN gene expression, albeit a recent study reported that WPI does not affect the weight of the epididymal tissue in HFD fed mice, but instead causes a reduction in subcutaneous fat pad weight [20]. These data suggest that WPI affects cellular activity in the liver and in specific adipose tissue depots. While it has been suggested that whey protein may facilitate enhanced postprandial chylomicron clearance via an alteration in LPL expression/activity [73], [74], here we did not find a WPI specific effect on LPL expression or plasma TAG levels, but we did observe that intake of the highest P/C ratio (40%-WPI) led to an increase adipose tissue LPL mRNA expression which was accompanied by significant reduction in plasma TAG levels, and a complete reversal of genes involved in lipid accumulation (PPARγ), fatty acid transport (FATP1), and lipolysis (β3-AR). Given that HF feeding/obesity down-regulates β3-AR mRNA expression [75], our data suggests an increased adipocyte lipolysis, and reduction in adipose TAG storage in HFD-40%WPI fed mice. Yet the endogenous CPT1b-associated β-oxidation pathway and the UCP-2-associated pathway in epididymal adipose tissue seem to be unaffected (CTP1b) or suppressed (UCP-2) by raising the P/C ratio. This data raises the possibility that the free fatty acids generated from the potentially increased availability of β3-AR in the adipose may have been re-directed for utilisation by other physiological processes active in HFD-40% group, possibly leading to the increased metabolic activity (VO_2_) observed in the animals. It is also noteworthy that WPI has been shown to increase faecal fat excretion compared to casein [5], which may have also contributed to the decreased plasma TAG and NEFA seen here with intake of the highest P/C ratio diet (HFD-40%WPI).

Given the link between HFD-induced obesity, low-grade inflammation and insulin resistance [76], [77], one could argue that the dramatic reduction in fat mass observed with the highest P/C ratio may underlie the effects on inflammatory markers in the adipose tissue (TNFα and CD68) and the hypothalamus (TNFα), along with simultaneous changes in expression of genes involved in insulin signalling (IR, IRS-1 and GLUT4 in the adipose, IRS-1 in liver and IR in the hypothalamus), and the reduction in plasma glucose in these mice. Improvements to insulin sensitivity with WPI have been reported previously [9], [78], but our data suggested that only an increased P/C ratio in the HFD facilitated improvements to insulin signalling pathway associated gene expression, particularly in the adipose, in parallel with reduced HOMA-IR values.

### Composition of gut microbiota

While many of the effects described above may be due to direct WPI or P/C ratio-host interactions, the effect of WPI and P/C ratio on the composition of the gut microbiota may also play an important role in adiposity and weight gain in these animals. Here, high throughput sequencing based analyses of faecal microbial populations revealed the clustering of the microbiota from animals in receipt of 30 and 40% WPI diets away from those in receipt of 20% kJ WPI or HFD-casein diets. Tranberg *et al* [20] recently suggested that the efficient absorption of dairy whey proteins in the small intestine may explain the absence of changes in the faecal microbiota. This may explain the clustering of the microbiota from animals fed 20% WPI or HFD-casein diets in our study. However, it is apparent that the high concentrations of WPI present in the 30 and 40% WPI diets employed in our study had a more profound effect, possibly due to additional whey proteins finding their way to the large intestine and/or the overall change in the P/C ratio in the diet. Consumption of the 30 and 40% WPI diets did not result in a shift in the microbiota toward that of the LFD animals and thus the effects on weight gain are not simply due to an overall conversion to a LFD-like microbiota. Specific taxonomic changes were also noted in response to the different diets. In all cases dietary WPI resulted in significant increases in *Lactobacillaceae*/*Lactobacillus* and decreases in *Clostridiaceae*/*Clostridium*. Increased proportions of *Lactobacillus* have previously also been observed in a study of individuals following a regime of calorie restriction and exercise [79]. However, in contrast, increased proportions of *Lactobacillus* have also been noted in HFD fed rats [80] and diet-induced obese mice [81]. While specific species of *Lactobacillus* have been associated with both lean and obese gut microbiota profiles and also to play a role in obesity and immune response regulation [82]–[84], due to the length of the 16S sequences generated and the high degree of sequence homology, we cannot assess changes in proportions of *Lactobacillus* at the species level. An increase in the proportions of *Bifidobacteriaceae/Bifidobacterium* was also observed in both the HFD-20% WPI and HFD-30% WPI compared to the HFD group. This result, combined with the aforementioned increases in *Lactobacillaceae/Lactobacillus*, mirror those reported by Sprong *et al* who suggest that whey proteins act as grow factors for certain species of bacteria by an amino acid composition mediated mechanism [19]. This pattern did not extend to the HFD-40% WPI group suggesting that, at these high protein levels, other factors are at play. Our observations are also consistent with previous findings that high proportions of the class *Clostridiales* are associated with the gut microbiota of animals fed a HFD [85], while fasting reduces the levels of *Clostridium* [86]. Notably, *Clostridiaceae* can produce short chain fatty acids as a product of their metabolism [87], which can play an important role in the regulation of immune cells and has been associated with inflammation and obesity [88]. These differences, as well as others in the *Proteobacteria Actinobacteria Deferribacteres* (phylum), *Desulfovibrionaceae Deferribacteraceae Veillonellaceae* (family), *Desulfovibrio* and *Mucispirillum* taxa in the HFD-20% whey protein relative to HFD controls (20% casein) are particularly notable as these reflect changes resulting from the specific presence of whey proteins in the diet, in place of casein, rather than changes in the P/C ratio. Changes in relative proportions may be attributed to (a) the ability of bacteria to utilise whey proteins as a growth medium, (b) the anti-microbial activity of whey protein/peptide components, (c) decreased competition as a result of the whey proteins/peptides antimicrobial activity or (d) whey protein mediated changes in the host. Ultimately, the question of cause versus effect remains unanswered, and so while the changes to the microbiota observed may contribute to the mechanisms involved in controlling weight gain, further studies with, for example germ free animals, will be required to determine this definitively.

### WPI effects on energy balance from a whole animal context

Focusing on the experimental data gathered between weeks 17–21, during which we measured metabolic parameters, faecal microbial population, body composition and tissue and plasma level of energy balance related parameters, it is clear that WPI intake increased energy intake associated with the HFD, without altering energy expenditure, as measured by VO_2_ and locomotor activity. However, the body composition and body weight in the HFD-WPI group does not appear to reflect a positive energy balance, as animals showed a trend towards a reduction in fat mass and increased lean mass. It is noteworthy in this regards that WPI has been shown to increase faecal fat excretion [5] and we also observed some subtle changes in the gut microbiota at a phylogenic level that are associated with non-obese states, raising the possibility of a reduced intestinal TAG absorption in the HFD-WPI groups with increased energy intake, leading to similar body weight trajectories as HFD controls. Increasing the P/C ratio by changing WPI from 30 to 40% did not alter energy intake but significantly accentuated energy expenditure with a concurrent dramatic change in physiology.

In summary, our results show that WPI specifically normalises energy intake, increases lean mass and causes a trend towards a reduction in fat mass associated with prolonged high fat feeding. Raising the P/C ratio had no effect on energy intake but augmented metabolic activity and beneficially altered gene expression profiles for lipid metabolism, inflammation and insulin signalling, particularly in the adipose tissue. High throughput analysis of gut microbiota revealed distinct changes in microbial populations with increased P/C ratio causing clustering of 30/40% WPI groups together and distinct from those of HFD and 20% WPI groups, but with specific phylogenetic differences existing between the latter groups. These data indicate that changes to P/C ratio have a dramatic effect on energy balance and the composition of gut microbiota distinct from that seen with changes to protein source. Future studies should focus on determining whether the effects demonstrated for highest P/C ratio are specific to the WPI content, a consequence of macronutrient change, or both.

## Supporting Information

Figure S1 **Effect of a 10%kJ low fat diet with 20%kJ casein (LFD) or 20%kJ whey protein isolate (LFD-WPI) on (A) body weight, (B) energy intake (C) oxygen consumption (VO_2_) and (D) locomotor activity, which were measured in individual mice at 9 minute intervals over a 24 hour period using TSE Phenomaster cages.**
(DOC)

Table S1 **The composition of the low fat diet (LFD), high fat diet (HFD) and HFD with 20% kJ, 30% kJ or 40% kJ whey protein isolate (WPI)^1^.**
(DOC)

Table S2 **Sequences of mouse specific primers used in real-time PCR analysis^1^.**
(DOC)
