# Supplementary material for: Protein Quality and the Protein to Carbohydrate Ratio within a High Fat Diet Influences Energy Balance and the Gut Microbiota In C57BL/6J Mice
Source: PLoS One. 2014 Feb 10;9(2):e88904. doi: 10.1371/journal.pone.0088904 (PMC3919831; doi:10.1371/journal.pone.0088904)
Supplement: Figure S1 — Effect of a 10%kJ low fat diet with 20%kJ casein (LFD) or 20%kJ whey protein isolate (LFD-WPI) on (A) body weight, (B) energy intake (C) oxygen consumption (VO2) and (D) locomotor activity, which were measured in individual mice at 9 minute intervals over a 24 hour period using TSE Phenomaster cages. (DOC) [file pone.0088904.s001.doc]

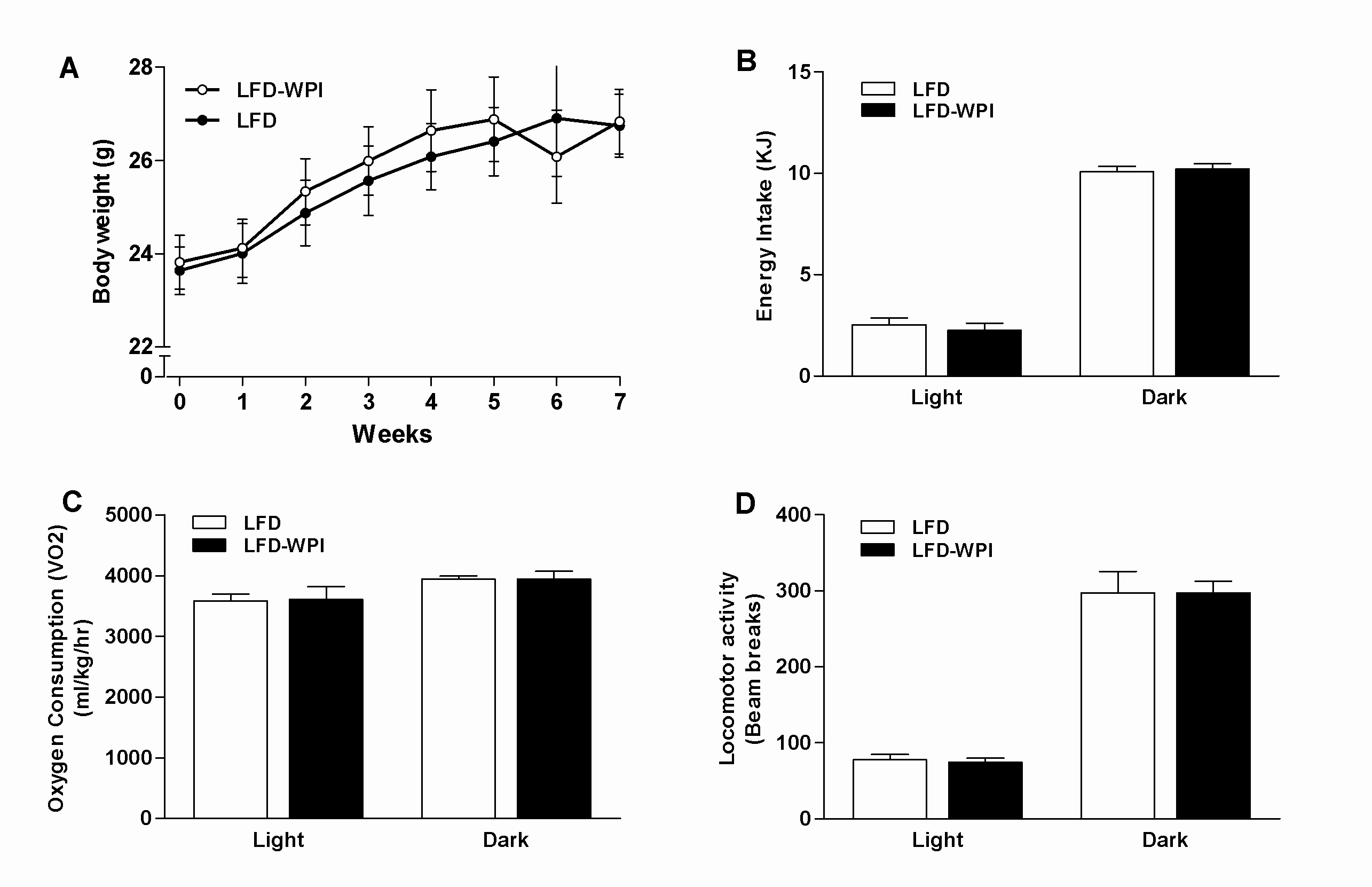


Figure S1: Effect of a 10%kJ low fat diet with 20%kJ casein (LFD) or 20%kJ whey protein isolate (LFD-WPI) on (A) body weight, (B) energy intake (C) oxygen consumption (VO_2_) and (D) locomotor activity, which were measured in individual mice at 9 minute intervals over a 24 hour period using TSE Phenomaster cages. Data are shown as mean values ± SEM (n = 8-10 per group) for light and dark phases.
