# Supplementary material for: Protein Quality and the Protein to Carbohydrate Ratio within a High Fat Diet Influences Energy Balance and the Gut Microbiota In C57BL/6J Mice
Source: PLoS One. 2014 Feb 10;9(2):e88904. doi: 10.1371/journal.pone.0088904 (PMC3919831; doi:10.1371/journal.pone.0088904)
Supplement: Table S1 — The composition of the low fat diet (LFD), high fat diet (HFD) and HFD with 20% kJ, 30% kJ or 40% kJ whey protein isolate (WPI)1. (DOC) [file pone.0088904.s002.doc]

**Table S1.** The composition of the low fat diet (LFD), high fat diet (HFD) and HFD with 20% kJ, 30% kJ or 40% kJ whey protein isolate (WPI) 1

| Contents (g) | Diets |  |  |  |  |
| --- | --- | --- | --- | --- | --- |
|  | LFD | HFD | HFD-20% WPI | HFD-30% WPI | HFD-40% WPI |
| Casein | 200 | 200 | 0 | 0 | 0 |
| Whey protein isolate | 0 | 0 | 200 | 300 | 400 |
| L-Cystine | 3 | 3 | 3 | 4.5 | 6 |
| Corn Starch | 315 | 72.8 | 72.8 | 47 | 0 |
| Maltodextrin 10 | 35 | 100 | 100 | 100 | 100 |
| Sucrose | 350 | 172.8 | 172.8 | 97.1 | 42.6 |
| Cellulose, BW200 | 50 | 50 | 50 | 50 | 50 |
| Soybean oil | 25 | 25 | 25 | 25 | 25 |
| Lard | 20 | 177.5 | 177.5 | 177.5 | 177.5 |
| Mineral mix S10026A | 10 | 10 | 10 | 10 | 10 |
| CaHPO_4_ | 13 | 13 | 13 | 13 | 13 |
| CaCO_3_ | 5.5 | 5.5 | 5.5 | 5.5 | 5.5 |
| C_5_H_6_K_3_O_7_.1H_2_O | 16.5 | 16.5 | 16.5 | 16.5 | 16.5 |
| Vitamin mix V10001 | 10 | 10 | 10 | 10 | 10 |
| Choline Bitartrate | 2 | 2 | 2 | 2 | 2 |
| Energy (kcal/g) | 3.8 | 4.7 | 4.7 | 4.7 | 4.7 |
| Protein (% kcal) | 20 | 20 | 20 | 30 | 40 |
| Carbohydrate (% kcal) | 70 | 35 | 35 | 25 | 15 |
| Fat (% kcal) | 10 | 45 | 45 | 45 | 45 |

^1^ Diets formulated and produced by Research Diets Inc. (New Brunswick, NJ, USA).
