# Supplementary material for: Protein Quality and the Protein to Carbohydrate Ratio within a High Fat Diet Influences Energy Balance and the Gut Microbiota In C57BL/6J Mice
Source: PLoS One. 2014 Feb 10;9(2):e88904. doi: 10.1371/journal.pone.0088904 (PMC3919831; doi:10.1371/journal.pone.0088904)
Supplement: Table S2 — Sequences of mouse specific primers used in real-time PCR analysis1. (DOC) [file pone.0088904.s003.doc]

Table S2. Sequences of mouse specific primers used in real-time PCR analysis^1^

|  | Forward primer (5’-3’) | Reverse primer (5’-3’) |
| --- | --- | --- |
| POMC | 5’-gggcaagcgctcctactccatg-3’ | 5’-ctcgccttccagctccctcttg-3’ |
| NPY | 5’-ccctcgctctatctctgctcgtgtg-3’ | 5’-gtagtatctggccatgtcctctgc-3’ |
| IR | 5’-gatttccccaacgtgtcctctac-3’ | 5’-caatgcggtacccagtgaagtg-3’ |
| IRS-1 | 5’-gcgcaggcaccatctcaacaacc-3’ | 5’-gcacgcacccggaaggaacc-3’ |
| GLUT4 | 5’-ggcctgcccgaaagagtc-3’ | 5’-aggagctggagcaaaggac-3’ |
| GLUT2 | 5’-tcctacttggcctatctgctgtgc-3’ | 5’-tgccctgacttcctcttccaac-3’ |
| ObR | 5’-gaccgccgaacacaaccgatgac-3’ | 5’-acacctagctggcgaaaaactgaag-3’ |
| GCCR | 5’-acctgacttccttgggggctatgaac-3’ | 5’-caggcagagtttgggaggtggtc-3’ |
| TNFα | 5’-tggcctccctctcatcag-3’ | 5’-acttggtggtttgctacgac-3’ |
| CD36 | 5’-tgatactatgcccgcctctcc-3’ | 5’-tttcccacactcctttctcctcta-3’ |
| β3-AR | 5’-cgccttcaacccggtcatctactg-3’ | 5’-ggtggactctgcctggcttcaac-3’ |
| PPARγ | 5’-tcaggtttgggcggatgc-3’ | 5’-tcagcgggaaggactttatgtatg-3’ |
| PPARα | 5’-atgggggtgatcggaggctaatag-3’ | 5’-gggtggcaggaagggaacagac-3’ |
| CPT1a | 5’-agacttccaacgcatgacagcactg-3’ | 5’-ctcggccccgcaggtagatg-3’ |
| CPT1b | 5’-cgagaggggcggactgagactg-3’ | 5’-ggctaggcggtacatgttttggtg-3’ |
| CPT1c | 5’-caggtgcgggagtcggtgaag-3’ | 5’-cagcagagcgtgggcataagc-3’ |
| FATP1 | 5’-ccggtgtggtggctgctcttctc-3’ | 5’-gctgccatctccccgccataaatg-3’ |
| FATP5 | 5’- ccggcagcatggcgtaacag-3’ | 5’-acacatttgcccgaagtccattg-3’ |
| FASN | 5’-tccacctttaagttgccctg-3’ | 5’-tctgctctcgtcatgtcacc-3’ |
| FABP1 | 5’-gaagcctcgttgccaccat-3’ | 5’-cgatttctgacacccccttgat-3’ |
| LPL | 5’-tgctcccaacaatataagactcc-3’ | 5’-aaggccaggtgtttcaatc-3’ |
| 11β-HSD1 | 5’-ccttggctgggaaaatgacc-3’ | 5’-ctatgaggccaaggacacagagag-3’ |
| UCP-2 | 5’-ccatttcctgcaccccgatttacttcc-3’ | 5’-gctgggctggggatgaagatgaag-3’ |
| GHS-R | 5’-ccccgggacaccaacgagtg-3’ | 5’-aagaccggcaggaagaagaagacg-3’ |
| Ghrelin | 5’-cagaaagcccagcagagaaaggaatc-3’ | 5’-cggccatgctgctgatactgag-3’ |
| CD68 | 5’-cacttcgggccatgtttctcttg-3’ | 5’-aggggctggtaggttgattgtcgtc-3’ |
| β-actin | 5’-agagggaaatcgtgcgtgac-3’ | 5’-caatagtgatgacctggcgt-3’ |
| GAPDH | 5’- ccattctcggccttgact-3’ | 5- tgaaggtcggtgtgaacg-3’ |
| 18-S | 5’-aggaccgcggttctattttgttgg-3’ | 5’-atgctttcgctct-ggtccgtcttg-3’ |
| YWHAZ | 5’-cggagctgcgtgacatctgc-3’ | 5’-cctcggccaagtaacggtagtag-3’ |

^1^ POMC, Pro-opiomelancortin; NPY, Neuropeptide Y; IR, Insulin receptor; IRS-1, Insulin receptor substrate 1; GLUT4, Glucose transporter 4; GLUT2, Glucose transporter 2; ; ObR, Leptin receptor; GCCR, Glucorticoid receptor; TNF-α, Tumour necrosis factor alpha; CD36, cluster of differentiation 36; β-3 AR, Beta 3 adrenergic receptor; PPARγ, peroxisome proliferator activated receptor gamma; CPT1a, carnitine palmitoyltransferase 1a, FATP1, Fatty acid transporter 1; FASN, Fatty acid synthase; FABP1, Fatty acid binding protein 1; LPL, Lipoprotein lipase; 11β-HSD1, 11β-hydroxysteroid dehydrogenase type 1; UCP-2, Uncoupling protein 2; GHS-R, Growth hormone secretagogue receptor; GAPDH, Gyceraldehyde 3-phosphate dehydrogenase.
